# Supplementary material for: In Vitro and In Silico Analyses Explore the Role of Flavonoid Classes in the Antiviral Activity of Plant Extracts Against the Dengue Virus
Source: Molecules. 2025 Nov 27;30(23):4566. doi: 10.3390/molecules30234566 (PMC12693615; doi:10.3390/molecules30234566)
Supplement: Supplementary file 1 [file molecules-30-04566-s001.zip › molecules-3968708-supplementary.pdf]

Article

In vitro and *in silico* analyses explore the role of flavonoid classes in the antiviral activity of plant extracts against the dengue virus

Sindi A. Velandia[1], Elena E Stashenko[1], Elizabeth Quintero-Rueda[1], Sergio Conde-Ocazionez[2], Lady J. Sierra[1], Raquel E Ocazionez[1],*

Table S1. Amount (mg/g) of compounds in extracts prepared in previous studies according to UHPLC/ESI-Q-Orbitrap-MS analysis. References 20-22.

**Name**	***S. coccinea***	***S. incarnata***	***L. origanoides* chemotypes**			
	**ScUA**	**SiUA-1**	**LopUA-1**	**LopSFE**	**LocSFE**	**LotSFE**
**Flavonoid glycosides**						
Apigenin-7-glucuronide	<LOD	<LOD	-	-	-	-
Baicalin	158.3 ± 0.3	278.1 ± 0.0	-	-	-	-
Dihydrobaicalein-glucuronide	51.2 ± 0.0	140.1 ± 0.00	-	-	-	-
Isocarthamidin-glucuronide	11.4 ± 0.0	13.11 ± 0.00	-	-	-	-
Eriodictyol-7-glucoside	-	-	3.5 ± 0.5	-	-	-
Eriodictyol-rhamnoside	-	-	0.61 ± 0.0	-	-	-
Phloridzin	-	-	0.26 ± 0.0	-	-	-
Galangin-glucoside	-	-	0.60 ± 0.0	-	-	-
Luteolin-7-glucoside	-	-	6.7 ± 0.9	-	-	-
Isoliquritin	1.5 ± 0.0	<LOD	-	-	-	-
Luteolin-rutinoside	-	-	0.12 ± 0.0	-	-	-
Norwogonin-glucuronide	<LOD	<LOD		-	-	-
Quercetin-3-glucoside	-	-	9.3 ± 1.3	-	-	-
Schaftoside	0.84 ± 0.0	0.83 ± 0.0	-	-	-	-
Scutellarin	21.4 ± 0.0	33.7 ± 0.0		-	-	-
Taxifolin-glucoside	-	-	0.12 ± 0.0	-	-	-
Wogonoside	1.2 ± 0.0	1.2 ± 0.0	-	-	-	-
**Flavonoid aglycones**						
Apigenin	-	-	-	<LOD	0.14 ± 0.0	0.44 ± 0.0
Baicalein	18.5 ± 0.0	16.4 ± 0.0	-	-	-	-
Chrysoeriol	-	-	0.07 ± 0.0	0.04 ± 0.0	1.3 ± 0.1	2.3 ± 0.1
Cirsimaritin	-	-	0.07 ± 0.0	0.05 ± 0.0	2.3 ± 0.0	3.5 ± 0.1
Dihydrobaicalein	8.8 ± 0.0	7.3 ± 0.0	-	-	-	-
Eriodyctiol	-	-	2.2 ± 0.1	0.44 ± 0.1	4.9 ± 0.2	3.6 ± 0.3
Galangin	-	-	0.40 ± 0.0	7.2 ± 0.2	<LOD	<LOD
Hesperetin	-	-	-	0.06 ± 0.0	0.77 ± 0.0	1.7 ± 0.0

Table S1. *Cont.*

Luteolin	-	-	0.93 ± 0.0	0.06 ± 0.0	0.46 ± 0.0	0.43 ± 0.0
Naringenin	-	-	0.11± 0.0	1.1 ± 0.0	4.6 ± 0.2	5.5 ± 0.6
Norwogonin	<LOD	<LOD	-	-	-	-
Phloridzin	-	-	0.26 ± 0.0	-	-	-
Pinocembrin	-	-	0.47 ± 0.0	48.3 ± 1.0	0.67 ± 0.0	2.0 ± 0.2
Quercetin	-	-	1.08 ± 0.0	0.16 ± 0.0	1.00 ± 0.0	<LOD
Sakuranetin	-	-	0.01 ± 0.0	1.42 ± 0.0	3.1 ± 0.3	4.8 ± 0.1
Scutellarein	3.8 ± 0.0	2.1 ± 1.0	-	-	-	-
Taxifolin	-	-	0.73 ± 0.0	0.06 ± 0.0	0.19 ± 0.0	0.20 ± 0.0
Wogonin	0.7 ± 0.3	0.16 ± 0.0	-	-	-	-
**Methylated flavonoids**						
Dimethylated flavone	-	-	-	-	0.23 ± 0.0	0.75 ± 0.0
Methylapigenin	-	-	-	-	0.15 ± 0.0	0.66 ± 0.0
Methylgalangin	-	-	0.10 ± 0.0	-	<LOD	<LOD
Trimethyltricetin	-	-	0.02 ± 0.0	-	-	-
**Non-flavonoids**						
Verbascoside	32.1 ± 0.0	33.7 ± 0.0	-	-	-	-
Umbelliferone-hexoside-pentoside	0.60 ± 0.0	0.423 ± 0.0	-	-	-	-

Extracts ScUA and SiUA-1 were prepared using the ultrasound-assisted solvent technique [25]. LopUA-1 (phellandrene chemotype) extract was prepared by ultrasound-assisted extraction [23], LocSFE (carvacrol chemotype) and LotSFE (thymol chemotype) were prepared by supercritical fluid extraction [24].

Table S2. Binding mode predicted for flavonoids found in the test extracts to targets. Proteins (PDB ID): Cl, clathrin N-terminal domain (2XZG); DENV-2 E, envelope (1OAN); DY, dynamin GTPase domain (2X2E); GX: Gas6-Axl receptor (2C5D).

Name	PubChem CID	Target: site	kcal/mol
Apigenin-7-glucoside 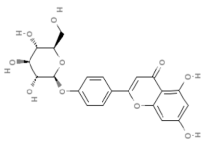	5491384	Cl: W-box	-9.36 ± 0.35
		DY: G4-Switch 1	-9.05 ± 0.43
		E: DII (A/B)	-8.32 ± 0.28
Isoliquiritin 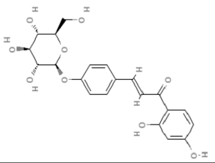	5318591	DY: G4-Switch 1	-9.19 ± 0.61
		Cl: W-box	-9.12 ± 0.33
		E: DII (A/B)	-8.03 ± 0.10
Norwogonin-glucuronide 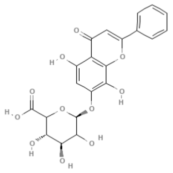	44258552	DY: P-loop	-8.85 ± 0.18
		Cl: W-box	-8.42 ± 0.32
		E: DII (A/B)	-8.18 ± 0.24
		GX: Gas6-Lg1/Lg2	-7.70 ± 0.11
Scutellarein 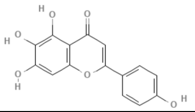	5281697	DY: Switch 1-P-loops	-8.18 ± 0.35
		Cl: W-box	-8.12 ± 0.46
		E: DII (A/B)	-7.59 ± 0.52
Norwogonin 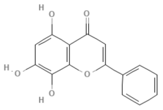	5281674	E: βOG pocket	-8.06 ± 0.80
		DY: Switch 2	-7.85 ± 0.54
		Cl: W-box	-7.79 ± 0.29
Apigenin* 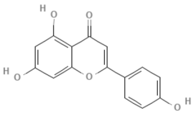	5280443	DY: G4-Switch 1	-8.02 ± 0.31
		Cl: W-box	-7.98 ± 0.41
		E: βOG pocket	-7.92 ± 0.60
Cirsimaritin*¶ 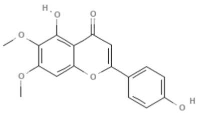	188323	Cl: W-box	-7.75 ± 0.43
Acacetin 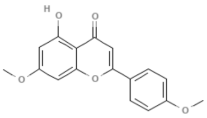	5281601	DY: G4-Switch 1	-8.18 ± 0.33
		Cl: W-box	-8.09 ± 0.38
		E: βOG pocket	-7.86 ± 0.40

Table S2. *Cont.*

Nepetin 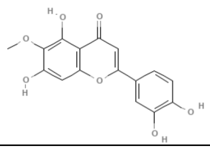	5317284	Cl: W-box<br>DY: G4-Switch 1<br>E: DII (A/B)	-8.12 ± 0.48<br>-8.01 ± 0.59<br>-7.55 ± 0.40
Salvigenin 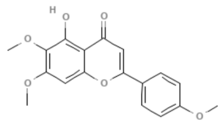	161271	Cl: W-box	-7.83 ± 0.38
Luteolin-rutinoside 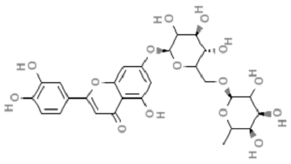	44258082	Cl: W-box<br>E: DII (A/B)<br>DY: Switch 1-P-loop<br>GX: Gas6-Lg1	-10.22 ± 0.34<br>-9.16 ± 0.18<br>-8.82 ± 0.13<br>-8.46 ± 0.25
Galangin-glucoside 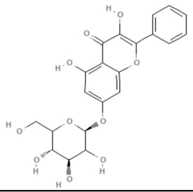	44258724	Cl: W-box<br>DY: G4-Switch 1<br>E: DII (A/B)	-8.70 ± 0.40<br>-8.27 ± 0.25<br>-7.82 ± 0.30
Eriodyctiol-7-glucoside 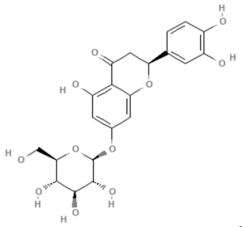	13254473	Cl: W-box<br>DY: G4-Switch 1<br>E: DII (A/B)<br>GX: Gas6-Lg1/Lg2	-8.67 ± 0.40<br>-8.16 ± 0.33<br>-8.08 ± 0.14<br>-7.77 ± 0.12
Quercetin-3-glucoside 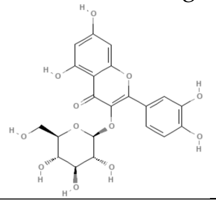	5280804	Cl: W-box	-8.89 ± 0.20
Luteolin 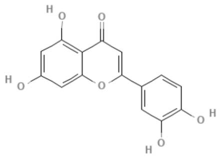	5280445	DY: G4-Switch 1<br>Cl: W-box<br>E: DII (A/B)	-8.23 ± 0.39<br>-8.22 ± 0.46<br>-7.96 ± 0.37

Data for the E protein was reported in a previous study [23].

Table S2. *Cont.*

Eriodyctiol-rhamnoside 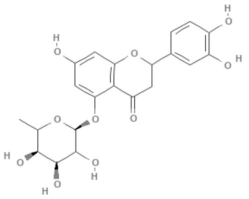	42607967	Cl: W-box	-8.22 ± 0.17
		E: DII (A/B)	-7.73 ± 0.45
		DY: Switch 1- P-loops	-7.68 ± 0.49
		GX: Gas6-Lg1/Lg2	-7.67 ± 0.34
Taxifolin-glucoside 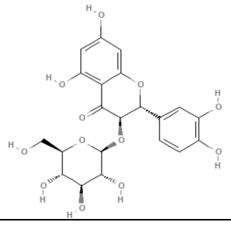	14187089	Cl: W-box	-8.19 ± 0.31
		DY: Switch 2- P-loops	-7.85 ± 0.26
Eriodyctiol 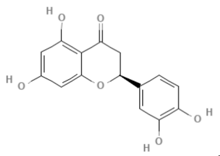	440735	Cl: W-box	-8.19 ± 0.51
		DY: G4-Switch 1	-8.11 ± 0.21
		E: βOG pocket	-8.05 ± 0.43
Phloridzin 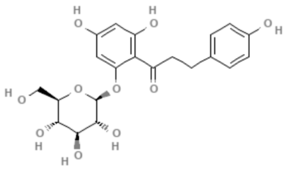	6072	DY: G4-Switch 1	-8.07 ± 0.53
		Cl: W-box	-7.87 ± 0.22
Pinocembrin 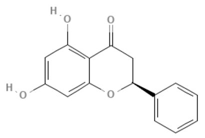	68071	E: βOG pocket	-8.00 ± 0.77
		Cl: W-box	-7.88 ± 0.44
		DY: Switch 2	-7.87 ± 0.36
Galangin 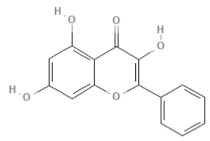	5281616	Cl: W-box	-7.88 ± 0.26
		DY: Switch 2- P-loops	-7.79 ± 0.31
Naringenin 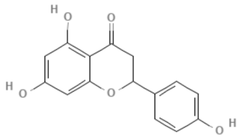	932	Cl: W-box	-7.96 ± 0.44
		E: βOG pocket	-7.94 ± 0.66
		DY: Switch 2- P-loops	-7.89 ± 0.23

Data for the E protein were reported in a previous study [23].

Table S2. *Cont.*

Quercetin<br>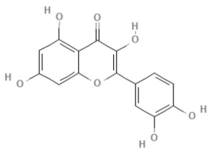	5280343	DY: Switch 2- P-loops<br>Cl: W-box	-8.14 ± 0.31<br>-7.93 ± 0.39
Sakuranetin<br>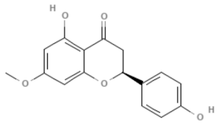	73571	Cl: W-box<br>DY: Switch 2- P-loops	-7.87 ± 0.45<br>-7.73 ± 0.35
Taxifolin<br>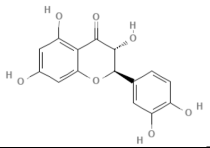	439533	DY: Switch 2- P-loops<br>Cl: W-box	-7.97 ± 0.31<br>-7.91 ± 0.44
Chrysoeriol¶<br>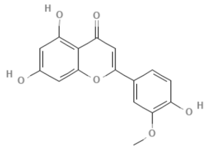	5280666	DY: G4-Switch 1<br>Cl: W-box	-8.19 ± 0.18<br>-7.90 ± 0.29
Hesperetin¶<br>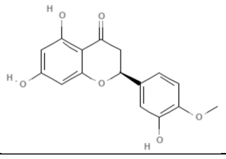	72281	Cl: W-box<br>DY: G4-Switch 1<br>E: DII (A/B)	-8.18 ± 0.48<br>-8.14 ± 0.34<br>-7.73 ± 0.33
Methyl-apigenin<br>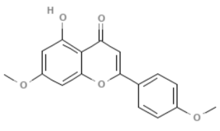	5281601	DY: Switch 1<br>Cl: W-box	-8.02 ± 0.28<br>-8.05 ± 0.37
Schaftoside<br>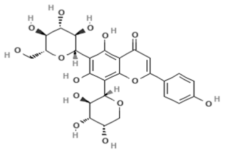	442658	DY: P-loop<br>Cl: W-box<br>E: DII (A/B)	-8.35 ± 0.34<br>-8.08 ± 0.25<br>-7.50 ± 0.26
Trimethyltricetin<br>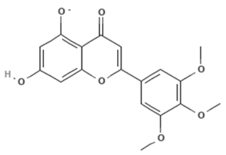	4687517	Cl: W-box	-7.51 ± 0.16

¶: Data for the E protein was reported in a previous study [23]. Methyl-galangin (CD:5488105) showed no binding affinity with any of the protein targets (with negative energy values greater than -7.5 kcal/mol).
